# The Week's Work

**Published:** 1910-05-21

**Authors:** 


### The Week's Work.

### ECHOES OF THE KING'S DEATH.

Last week there was only one event that exercised the mind of the public. This week, although, in a sense, the King's death has lost its importance as a subject for discussion, it is very evident that in the hospital world at least its influence is still widely felt. The postponement of all hospital functions was a matter of course. We know of no institution that has persisted with its prearranged programme. All have loyally and sympathetically sacrificed their interests for the moment to show that they truly respected the memory of one who was so sincerely their wellwisher in the past and that they mourned in sympathy with one who has shown himself so pre-eminently their friend and supporter. That was only as it should have been. No hospital will in any way be the worse for that sacrifice; many in some ways are already the better for it. The public is slowly beginning to realise how dominant was the interest which King Edward and Royalty generally took in hospital affairs, and it is possible that the example which, while his late Majesty was alive, so actively stimulated the public sense of generosity and charitableness, will be no less effective now that the King is dead.

### INTEREST IN HOSPITAL WORK.

When it is possible that the gaieties and distractions of a London season will, if not wholly curtailed, at least be abrogated, there is reason for believing that a more general and a more generous interest in charitable work will animate the public, and that the hospitals will largely benefit by that spirit. Here and there its beginnings may be noted. Several institutions have received gifts from persons not on their regular subscribers' lists as donations forwarded in memory of King Edward VII., and we fully expect that the number and amount of such voluntary donations will be largely increased during the coming months. No hospital secretary can afford not to utilise the opportunity now afforded him to secure new friends and subscribers. Some of those who have forwarded gifts have done so simply because they have become acquainted with the fact that the late King was a patron of the special institution to which they have sent a cheque. Such subscribers can be retained and turned into permanent friends of the charity if the needs of the institution are forcibly represented to them, and there is no time like the present for doing so. Large hospitals with which the late King was prominently identified may find it in their interest to issue a special report, pointing out his late Majesty's interest in the charity and what he did for it and how liberally he supported it. By so doing they will at least bring before the public once more the stimulating example of Royalty which is bound to exert its influence upon the public, and in any case they will make their institutions known to a wide circle. We throw out the suggestion in the hope that some institutions may profit by it.

### LOCAL KING'S FUNDS.

An interesting suggestion of another sort comes from overseas. The Archbishop of Cape Town, speaking his eulogy upon King Edward, drew attention to the fact that the King had been specially interested in the establishment of the Prince of Wales Hospital Fund for London, and suggested that one of the best means of perpetuating his Majesty's memory would be by the establishment of a similar fund, to be known, as the Prince of Wales' Fund is now known, as King Edward VII.'s Fund for Hospitals. The suggestion, we are glad to note, has found favour in South Africa, where, it must be remembered, hospitals already receive a grant from Government, which in some respects fills the place which in London is filled by the King's Fund. Incidentally, it must also be borne in mind that the hospital system in South Africa is, in a sense, in a state of transition. The advent of Union has brought about a desire in the new dominion for a reformation in the medical profession, or at least a modification of the various laws that affect medical men, and for more particularly and clearly defining their relations to the State. It is quite probable that such a codification will sensibly react upon the hospital world and that the present rather unsatisfactory relations between colonial hospitals and the Government will have to be revised. In the circumstances, the suggestion thrown out by the Archbishop is perhaps less likely to be immediately conducive to the establishment of a large central fund in South Africa than it is in England and the other dominions. There is no reason why there should not be a King's Fund in every large city in the Empire. The arguments against such a central fund have long been threshed out, and have been found to be valueless so far as the practical utility of central organisation is concerned. If instead of one central London fund there were in existence corresponding funds in all the big cities where hospital needs are specially prominent, we should hear little of the demerits, from a financial point of view, of the voluntary system. The establishment of such local funds would go far to promote an interest in the welfare of hospitals and charitable institutions, and would no more clash with the periodical collections in aid of the sister funds than does the King's Fund with the London collections in aid of the Saturday and Sunday Funds. In this respect the example of the League of Mercy, with its manifold local activities, is one that may usefully be imitated.

### RULERS AND THEIR HOSPITALS.

The interest that the late King took in hospital affairs was exceptional and perhaps unequalled by any other modern ruler. Nevertheless it is worth while to consider what contemporary sovereigns are doing to help the cause of such institutions. And first of all stands the noticeable fact that the heads of Republics appear to interest themselves comparatively little in the welfare of hospitals. The present President of the French Republic, for instance, though he has opened several hospitals, is not a familiar figure to hospital workers in his own country, while President Taft is no exception to the Republican rule. Certainly neither of them can compete with the late King as a personal worker on behalf of hospital charities. The Queen of Holland and her Consort are well known for their interest in charitable work. So, too, are the rulers of Italy, Denmark, Sweden, Norway, and Spain. King Leopold often visited hospitals and spent some time in conversation with the patients—excursions which he is said to have enjoyed as much as his walks around the markets—and his successor has inspected more than one institution. The Emperor Francis Joseph has always taken a keen interest in military hospitals and lazarettos, and in former years, when his activities were greater than at present, he was in the habit of visiting informally many of the large hospitals which are among the proudest assets of Austria, while his interest in the construction of new buildings was not merely that of a bored spectator. Of King Ferdinand and King Carlos comparatively little is known as hospital workers, but it can scarcely be questioned that they, too, have been hospital visitors.

### THE TSAR AND THE KAISER.

But the only monarchs who can be said to have vied with King Edward in their hospital activities are the German Emperor and the Emperor of Russia. The latter is an indefatigable hospital visitor, and the excellence of the Russian institutions, which is the admiration and astonishment of everyone who travels in that interesting empire and takes the trouble to inspect its institutions, is undoubtedly mainly due to the keen and active practical interest that the Imperial family, from the Tsar downwards, has always taken in hospital affairs. The Emperor is, we believe, the patron of all the Russian hospitals, and similarly their supporter. He makes surprise visits every year, and displays as eager an interest in the management and affairs of the charity he visits as King Edward did. Similarly the Kaiser has always taken a practical interest in the hospitals of the German Empire. When the Rudolph Virchow Hospital at Berlin was being built his Imperial Majesty inspected the site, and the plans were submitted to him and modified in some degree to meet his wishes. Since it is in working he has on several occasions visited it informally, and has inspected it from top to bottom. His influence has also been felt in other towns where new hospitals were built, and he has made himself as familiar a figure in the German hospital as he is in the German medical world, where his interests in practical and scientific medicine are well known. On his visits abroad he and the Kaiserin have always made a point of visiting the local German hospital where such existed. He is one of the patrons of the German Hospital in London and of the German Hospital at Jerusalem, both of which he or the Kaiserin has visited on more than one occasion.

### AN ARMY HOSPITAL AFFAIR.

In the *Regiment* of the 16th ult. prominence is given to what is styled an "Army hospital scandal." It is stated that at the military hospital at Bodmin, Cornwall, the medical officer is unpunctual and irregular in attendance, and that, for instance, a patient was not seen for a week following his admission. The supply of medical extras in the way of diet is alleged to be uncertain. Dissatisfaction is also expressed with the employment of military patients about the hospital and its grounds in tasks like grooming, gardening, or office duty, it being stated that men with the most trivial ailments are brought regularly to hospital for participation in unpleasant labour to the neglect of their more legitimate military training. The combatant authorities are stated to have objected in two instances to occurrences of this nature. Finally, an editorial note appended mentions the arrival of a "senior officer" at Bodmin to open investigations. The above would seem to warrant our taking cognisance of these statements here, although perusal of a subsequent issue—that for April 30—of the same journal inclines us to think that very likely the real grievance will be found to be connected with the employment of military hospital patients. Thus a R.A.M.C. non-commissioned officer stationed in Ireland writes rather indignantly to say that work for slight cases of illness such as are usually found in military hospitals is an excellent thing, and serves *pour encourager les autres*—that is, it discourages malingering to avoid military duty. Moreover, what in another column is given the opprobrious name of the "blacklead-and-scrubber cure," is stated to be in use not only at Bodmin, but also at Hounslow and Colchester. And we should think better of our contemporary's good judgment if it had not, a little lower down the page, censured an unlucky infantry colonel for rewarding men who had submitted to anti-typhoid inoculation. But, of course, such charges of negligence as have been made demand the strictest investigation. We believe there is only one military medical officer at Bodmin, a lieutenant-colonel of the R.A.M.C. on half-pay.

### THE DRUG MARKET.

Business has been more or less at a standstill since the last report appeared, and few changes of any particular note in the price of drugs have taken place. Some business has been done in jalap at good prices, and as the stock in London is small, values seem more likely to advance than recede. Cod-liver oil is still tending lower in price, mainly owing to the smallness of the demand and to the fact that the result of the fishing has latterly been more satisfactory. Extremely high prices are still asked for cubebs. The outlook regarding the new opium crop in Smyrna is very favourable, and buyers are not anxious to purchase at the present rates.

